# BP1003 Decreases STAT3 Expression and Its Pro-Tumorigenic Functions in Solid Tumors and the Tumor Microenvironment

**DOI:** 10.3390/biomedicines12081901

**Published:** 2024-08-20

**Authors:** Maria Gagliardi, Rhonda Kean, Bingbing Dai, Jithesh Jose Augustine, Michael Roberts, Jason Fleming, D. Craig Hooper, Ana Tari Ashizawa

**Affiliations:** 1 Bio-Path Holdings Inc., Bellaire, TX 77401, USA; mgagliardi@biopathholdings.com (M.G.);; 2 Department of Cancer Biology, Philadelphia, Thomas Jefferson University, PA 19107, USA; 3 Department of Surgical Oncology, The University of Texas MD Anderson Cancer Center, Houston, TX 77030, USA; 4 Independent Researcher, Houston, TX 77030, USA

**Keywords:** STAT3, antisense oligodeoxynucleotide, breast, ovarian, pancreatic cancer, monocyte polarization, M2 macrophages

## Abstract

Overexpression and aberrant activation of signal transducer and activator of transcription 3 (STAT3) contribute to tumorigenesis, drug resistance, and tumor-immune evasion, making it a potential cancer therapeutic target. BP1003 is a neutral liposome incorporated with a nuclease-resistant P-ethoxy antisense oligodeoxynucleotide (ASO) targeting the STAT3 mRNA. Its unique design enhances BP1003 stability, cellular uptake, and target affinity. BP1003 efficiently reduces STAT3 expression and enhances the sensitivity of breast cancer cells (HER2^+^, triple negative) and ovarian cancer cells (late stage, invasive ovarian cancer) to paclitaxel and 5-fluorouracil (5-FU) in both 2D and 3D cell cultures. Similarly, ex vivo and in vivo patient-derived models of pancreatic ductal adenocarcinoma (PDAC) show reduced tissue viability and tumor volume with BP1003 and gemcitabine combination treatments. In addition to directly affecting tumor cells, BP1003 can modulate the tumor microenvironment. Unlike M1 differentiation, monocyte differentiation into anti-inflammatory M2 macrophages is suppressed by BP1003, indicating its potential contribution to immunotherapy. The broad anti-tumor effect of BP1003 in numerous preclinical solid tumor models, such as breast, ovarian, and pancreatic cancer models shown in this work, makes it a promising cancer therapeutic.

## 1. Introduction

Overexpression and persistent activation of STAT3 have been correlated with poor patient prognosis in various types of cancers (including breast, ovarian, pancreatic, lung, and colon cancer). This is likely because STAT3 dysfunction contributes to cancer cell proliferation, survival, drug resistance, metastasis, and immune evasion [1,2,3]. Preclinical studies show that activation of the JAK-STAT3 pathway in triple negative breast cancer (TNBC) or the FGFR1/SRC/STAT3 pathway in ovarian cancer promotes tumor initiation, migration, and paclitaxel resistance [4,5,6,7]. STAT3 also induces epithelial–mesenchymal transition in hepatocellular carcinoma and attenuates the cell’s response to 5-FU [8]. The predominant role of STAT3 in pancreatic cancer is the promotion of angiogenesis via enhanced expression of vascular endothelial growth factor [9,10].

In addition to its intrinsic pro-tumoral roles, STAT3 mediates immune suppression in the tumor microenvironment [11,12,13]. Recent studies in a TNBC mouse model show that inhibition of STAT3 not only reduces the elevated levels of PD-L1 expression, which serves to bind and inhibit T cell function [14], but also directs the phenotype of tumor associated macrophages (TAM) towards an anti-tumorigenic M1 phenotype [15].

Contrary to the M1 phenotype, TAM polarized to an M2 phenotype create a pro-tumorigenic environment by suppressing CD8^+^ T and NK cell functions through the production of anti-inflammatory factors and by releasing growth and angiogenic factors that support tumor growth and progression [16,17]. M2 polarization is dependent on STAT3 signaling which is often directly elicited by cancer cells [18]. For example, recent work has shown that the release of lactate from TNBC cells or chemokine ligand 2 (CCL2) from ovarian cancer cells induces the chemotaxis of macrophages, STAT3 signaling, and M2 differentiation [19,20,21]. Immune cell function can also be modulated by cancer-derived exosomes containing STAT3 activating cargo. Pancreatic cancer derived exosomes containing lncRNA FGD5-AS1 stimulate STAT3 acetylation and transcriptional activity in surrounding monocytes, promoting M2 polarization [22].

Being a point of convergence of multiple upstream tyrosine kinases (both receptor and non-receptor) and being regulated by a range of post-translational modifications and noncoding RNA make indirect targeting of STAT3 insufficient, with compensatory pathways taking over and off target inhibition of other STATs. A range of direct STAT3 inhibitors targeting its SH2 and DNA binding domains are currently being evaluated in pre-clinical studies and ongoing clinical trials [12,23,24]. Although small molecule and peptide inhibitors are promising, antisense oligonucleotide (ASO) technology has the advantage of specifically blocking the synthesis of the targeted protein with more stability and less potential toxicity [25].

BP1003 consists of a nuclease-resistant P-ethoxy antisense oligodeoxynucleotide (DNAbilize^®^ technology) targeting the STAT3 mRNA, which is incorporated in a non-immunogenic, neutral dioleoylphosphatidylcholine (DOPC) liposome. This innovative design makes BP1003 nuclease resistant and hydrophobic, thereby rendering it more stable and with enhanced cellular uptake. The benefits of the DNAbilize^®^ technology have been shown in the preclinical and clinical studies of prexigebersen and BP1002 which have proven to be very well tolerated with little to no toxicity in animal and clinical studies [26,27]. To date, danvatirsen (IONIS-STAT3/AZD9150) is the only available STAT3-ASO in clinical trial [24]; its clinical efficacy has yet to be established.

Here we report the effects of BP1003 as a single agent and in combination with chemotherapy in various cancer models. Our findings show that BP1003 significantly reduced the expression of STAT3 protein and that of its downstream targets, vimentin and Bcl-2. Vimentin has critical roles in maintaining cell integrity, epithelial mesenchymal transition, and lamellipodia formation [28,29,30]. Anti-apoptotic Bcl-2 protein is a prominent therapeutic target for hematological and various solid tumor malignancies [31,32,33]. The inhibitory effects of BP1003 on cell viability, motility, and spheroid growth were augmented in combination with paclitaxel or 5-FU. Additionally, the combination of BP1003 and gemcitabine reduced tumor viability and promoted tumor regression in ex vivo and in vivo pancreatic ductal adenocarcinoma (PDAC) patient-derived xenograft (PDX) models. BP1003 also proved highly effective at blocking the polarization of monocytes to a CD206+ M2 phenotype with no effect on the M1 phenotype in vitro.

## 2. Materials and Methods

### 2.1. Cell Culture

BT549, SK-OV-3, and SK-BR-3 cells, purchased from ATCC (Manassas, VA, USA)*,* were cultured in RPMI-1640 medium (GenDEPOT, Katy, TX, USA) supplemented with 10% fetal bovine serum (FBS, GenDEPOT) and penicillin–streptomycin (100 units/mL, GenDEPOT) in a 37 °C humidified incubator under 5% CO_2_. Cells were passaged every 3 days. Cell lines were authenticated prior to freezing for long term storage and periodically when in culture.

### 2.2. BP1003

The STAT3 ASO (5′-CTG ATA ATT CAA CTC AGG-3′) was manufactured by Nitto Denko Avecia Inc. (Cincinnati, OH, USA). DOPC lipids were purchased from Avanti Polar Lipids, Inc. (Alabaster, AL, USA). STAT3 ASO was formulated with DOPC lipids and lyophilized in a LyoStar 3 lyophilizer (SP Industries, Inc., Warminster, PA, USA). Drug vials were stored at 2–8 °C. Prior to use, BP1003 vials were acclimated to room temperature before being reconstituted with sterile phosphate buffer saline (PBS) (GenDEPOT). The vials were then vortexed at 2500 rpm for 2 min at room temperature.

### 2.3. Viability Assay

Cell viability was quantified using an alamarBlue (BioRad, Fort Worth, TX, USA) viability assay. In brief, 1200 BT549, 2000 SK-OV-3 or 2000 SK-BR-3 cells, suspended in 100 µL of complete medium, were plated in 96-well plates and treated with 100 µL of complete medium containing paclitaxel or 5-FU (Caymen Chemical, Ann Arbor, MI, USA). Four hours later, BP1003 or empty liposomes (EL) were added to the wells at a final concentration of 200 µg/mL. After 96 h of incubation, resorufin’s red fluorescence was quantified in a FLUOstar microplate reader (BMG Labtech, Cary, NC, USA) using 560/590 nm (excitation/emission) filters.

### 2.4. Immunoblotting

Cells were lysed in RIPA lysis buffer (VWR Life Science, Missouri, TX, USA) containing protease and phosphatase inhibitors (Bimake, Houston, TX, USA). Protein lysates were separated by SDS-PAGE (Thermo Fisher Scientific, Waltham, MA, USA), transferred to nitrocellulose membranes (Bio Rad, Hurcules, CA, USA), blocked with 4% non-fat dry milk (ChemCruz, Dallas, TX, USA) in 1× Tween-20 TBS (wash buffer) (ChemCruz) for 1 h at room temperature, and followed by an overnight incubation at 4 °C with primary antibody (4% (*w*/*v*) milk in wash buffer). The following day, after three 10 min washes in wash buffer, membranes were incubated with the appropriate horseradish peroxidase (HRP)-conjugated secondary antibody for 2 h at room temperature, washed, and developed using enhanced chemiluminescence (ECL) (Thermo Fisher Scientific) in an Azure c300 imaging system (Azure Biosystems, Dublin, CA, USA). The primary antibodies used were anti-STAT3 (1:1000 dilution, BD Biosciences, Franklin Lakes, NJ, USA), anti-STAT4 (1:1000, Santa Cruz Biotechnology, Dallas, TX, USA), anti-Bcl-2 (1:500, Abcam, Waltham, MA, USA), anti-vimentin (1:1000, Cell Signaling, Danvers, MA, USA), and anti-β-actin (1:5000, Cell Signaling). Secondary antibodies for chemiluminescent signal detection were horseradish peroxidase-conjugated IgG (1:10,000 dilution; Rabbit: Cell Signaling or Mouse: Azure Biosystems). Band intensity was analyzed using ImageJ (version 1.54f).

### 2.5. Colony Formation

Five hundred SK-OV-3 or BT549 cells were seeded in triplicates in 6-well plates. After 24 h, cells were treated with 150 µg/mL BP1003 and 0.12 nM paclitaxel and incubated at 37 °C in a 5% CO_2_ humidified incubator. After 10 days, colonies were fixed in 3% (*v*/*v*) acetic acid, 10% (*v*/*v*) methanol solution for 2 min, and stained with 0.2% (*w*/*v*) crystal violet staining solution (Sigma, Burlington, MA, USA) for 20 min. Once washed and dried, colony number and optical densities were acquired in a FLUOstar microplate reader (BMG Labtech) at 590 nM wavelength.

### 2.6. Cell Migration

Migration assays were performed using 24-well micro-chemotaxis chambers (VWR). Cells were pre-treated with 200 µg/mL of BP1003 or EL for 72 h before being washed with PBS and incubated for 4 h in FBS-free medium with or without paclitaxel (2 nM, 2 nM, and 1.5 nM for SK-OV-3, SK-BR-3 cells, and BT549 cells, respectively). Cells were then seeded into upper chambers at a density of 1 × 10^5^ cells in 250 µL of FBS-free medium containing 200 µg/mL BP1003 or EL, in the presence or absence of paclitaxel. The lower chambers were filled with 650 µL complete medium supplemented with 10% FBS as an attractant. SK-OV-3 cells were given 8 h to migrate, BT549 cells were given 6 h and SK-BR-3 cells were given 20 h to migrate. Migrated cells were fixed and stained with 0.2% (*w*/*v*) crystal violet (Sigma) staining solution. Images of cells were captured by a Leica DFC camera on a Leica DM IL LED microscope (10× magnification). The percentage of membrane area taken up by migrated cells was determined using ImageJ.

### 2.7. Spheroids

Spheroids were formed in 96-well round bottom, ultra-low attachment plates (Corning) in 100 µL complete medium (500 SK-OV-3 cells, 250 BT549 cells). Plates were incubated at 37 °C in a 5% CO_2_ humidified incubator. Three days after plating, spheroids were treated with 250 µg/mL of BP1003 or EL, followed by 6 nM paclitaxel 24 h later for combination treatments. Spheroids were imaged using a Leica DFC camera on a Leica DM IL LED microscope (20× magnification) and analyzed using ImageJ. Larger BT549 and SK-OV-3 spheroids (formed from 4000 cells) were treated with 1 mg/mL BP1003. Since a high dose of BP1003 obstructed spheroid visibility, spheroids were transferred and gently washed in a 6-well plate containing 3 mL of PBS prior to imaging.

The effect of BP1003 on spheroid formation was assessed by pre-treating cells with 200 µg/mL of BP1003 or EL for 72 h. Cells were then removed from BP1003 and seeded in 96-well low-attachment round-bottom plates. One day after seeding, cells were treated again with 100 µg/mL of BP1003 alone or in combination with 5 nM paclitaxel. Growth kinetics was monitored for 9 days by measuring the area of the spheroid.

### 2.8. Ex Vivo LTSA Assay

PDAC PDXs were established and grown in immunodeficient mice as previously described [34]. Tissue cores (3 mm × 200 µm) were generated as previously described from a panel of 18 PDAC patient-derived xenografts [34]. Tissue slices were treated with BP1003 (60 µg/mL) and/or gemcitabine (10 µM). After 72 h, PrestoBlue^®^ reagent was added to the tissue slice culture medium for an additional 2 h. Fluorescent intensity was determined in a CLARIOstar^®^ plate reader (BMG LABTECH).

### 2.9. PDX In Vivo Experiment

To assess drug efficacy, a previously described animal experimental protocol was applied, which involves the implantation of ~10 mm^3^ tumorgrafts in nude mice [34]. Once tumors reach the size of ~100 mm^3^, mice were administered BP1003 (25 mg/kg) and/or gemcitabine (50 mg/kg) twice a week for 4 weeks. Tumor volumes were measured weekly. Immunohistochemistry, described in Roife et al. [34], was used to assess STAT3 protein levels in tumor tissues.

### 2.10. Preparation of Monocytes from Peripheral Blood

Human peripheral blood mononuclear cells (PBMC) were isolated from the blood of healthy adult volunteers obtained from Thomas Jefferson University Hospital blood bank. Buffy coats diluted 1:1 in PBS/2% FBS were processed using Lymphoprep (Stem cell technologies, Vancouver, BC, Canada) density gradient cell isolation according to manufacturer’s instructions. CD14+ cells were isolated from PBMC by positive selection using Miltenyi Biotec magnetic beads according to the manufacturer’s protocol.

### 2.11. Monocyte/Macrophage Culture Conditions

CD14+ cells were cultured at 2 × 10^6^/mL in Stemcell SF macrophage expansion medium overnight at 37 °C. The following day, CD14+ cells were diluted to 1 × 10^6^/mL with macrophage expansion medium containing 5 mg/mL human recombinant M-CSF. Cultures were activated with 10 ng/mL LPS and 50 ng/mL IFN-γ for M1 polarization or 10 ng/mL IL-4 for M2 polarization. Treatment during polarization: BP1003 and EL were added to cultures at 500, 250, and 125 µg/mL. Cells were incubated for 3 days at 37 °C, 5% CO_2_, then harvested for analysis by flow cytometry. The treatment after polarization: after 3 days of polarization, media was removed from wells and fresh macrophage media with 5 mg/mL M-CSF was added to cultures. BP1003 and EL were added to the cultures at 500, 250, and 125 µg/mL. Cells were incubated for 3 days at 37 °C, 5% CO_2_ and analyzed by flow cytometry.

### 2.12. Macrophage Phenotypic Analysis

Following monocyte/macrophage treatments, cells were resuspended in 100 mL of one of the antibody panels listed below containing a 1:100 dilution of each antibody in FACS buffer. Cells were incubated with FACS buffer only (no stain control) or with antibodies at 4 °C for 1 h, then washed and resuspended in 200 mL FACS buffer for analysis on a Guava flow cytometer. The antibodies used were CD14 (FITC, BD Biosciences #557153), CD86 (PE, BD #555658), CD206 (APC, BD #550889), CD16 (APC-Cy7, BD #557758), CD11B (FITC, Miltenyi #130-113-234), CD68 (PE, BD #556078), CD163 (PerCP, BD #563867), HLA-DR (APC, BD #559866), CD204 (PE, BD #566251), CD15 (FITC, BD #560997), CD14 (PE, BD #555398). Stain profiles were analyzed by FlowJo using singly stained beads for compensation where appropriate.

### 2.13. Statistical Analysis

Statistical analyses were performed on Graphpad Prism 9. Data were presented as means ± standard deviations (SD) or standard error of mean (SEM) as noted in figure legends. Significance was determined using Student’s unpaired *t*-test. Statistical significance was indicated with * *p* < 0.05, ** *p* < 0.01, and *** *p* < 0.001.

## 3. Results

### 3.1. BP1003 Reduces STAT3 Expression

**Figure 1:**
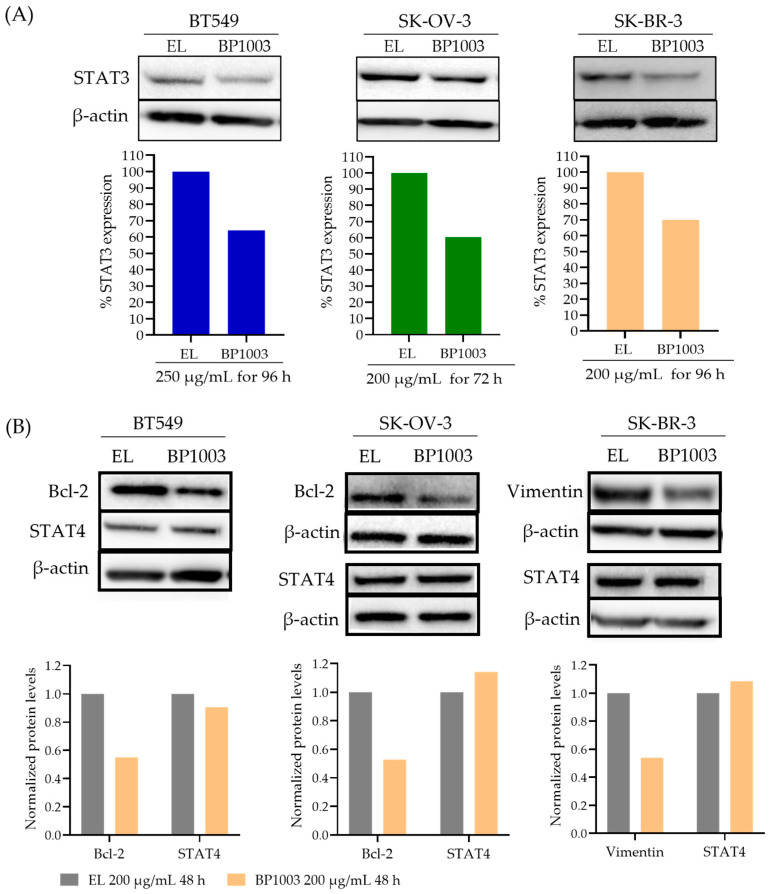
Effect of BP1003 on STAT3 and downstream proteins levels. (**A**) Representative immunoblots and normalized densitometric levels of STAT3 in BT549, SK-BR-3, and SK-OV-3 cell lines treated with BP1003 or EL. (**B**) Representative immunoblots and normalized densitometric levels of STAT4 and STAT3 target proteins, Bcl-2 and vimentin, after treatment with BP1003 or EL.

The ability of BP1003 to reduce the expression of STAT3 protein was assessed in human cancer cell lines. Treatment of BT549 cells (TNBC), SK-BR-3 cells (HER2+ breast cancer), and SK-OV-3 cells (late stage, invasive ovarian cancer) with 200 µg/mL of BP1003 for 96 h resulted in a 30–40% decrease in STAT3 protein levels compared to cells treated with EL (Figure 1A). Time course experiments showed a reduction in STAT3 expression as early as 48 h after treatment with a progressive decrease for 96 h (Figure S1A).

Due to target sequence similarity between STAT3 and STAT4 (Figure S1B), the effect of BP1003 on STAT4 levels was assessed. STAT4 protein levels were either unchanged or increased by BP1003 treatment, confirming the specificity of BP1003 for STAT3 (Figure 1B and Figure S1A). BP1003 also reduced the expression of STAT3 downstream targets, such as the anti-apoptotic Bcl-*2* protein in BT549 and SK-OV-3 cell lines (Figure 1B). Unlike STAT3 levels that continue to decrease with prolonged BP1003 exposure, Bcl-2 levels are stabilized after an initial decrease (Figure S1A). This could be due to additional regulators of Bcl-2 expression and protein stability. BP1003 also decreased the expression of the metastasis promoting vimentin protein, another STAT3 downstream target, in the SK-BR-3 cell line (Figure 1B). Effects of BP1003 on vimentin were assessed in SK-BR-3 cells because Bcl-2 protein level is very low in these cells. These immunoblots clearly show that BP1003 significantly reduces the protein levels of STAT3 and its downstream targets, which play critical roles in cancer progression.

### 3.2. BP1003 Reduces Cell Viability and Colony Formation in Combination Treatments

An alamar blue viability assay was used to determine the effect of BP1003 as a monotherapy and in combination with two widely used chemotherapeutic agents: paclitaxel and 5-FU. BP1003 decreased the viability of SK-OV-3, BT549, and SK-BR-3 cells in a dose-dependent manner (Figure S2A).

**Figure 2:**
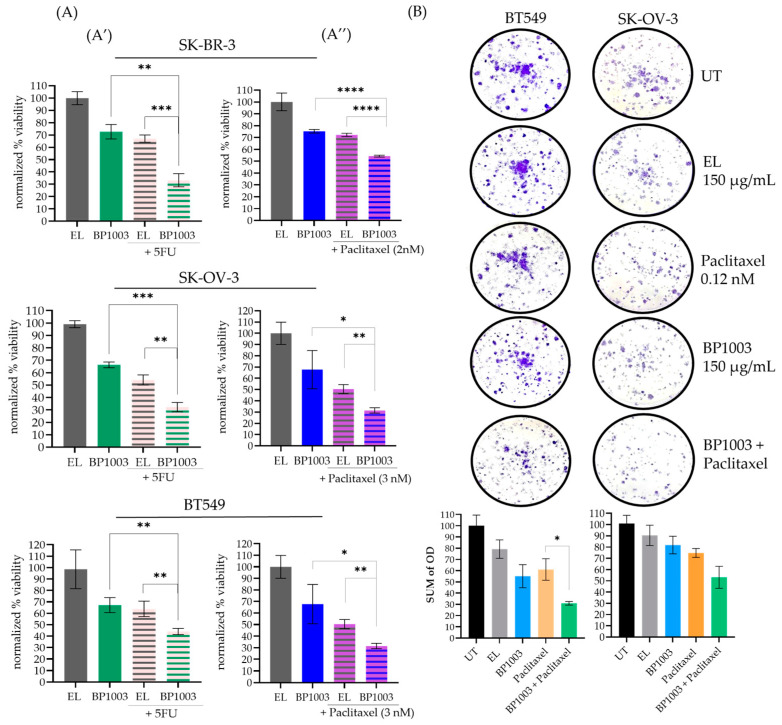
BP1003 reduced cell viability and colony formation. (**A**) Alamar blue viability assays were performed on SK-BR-3, SK-OV-3, and BT549 cells treated with BP1003 monotherapy at 200 μg/mL or in combination with 5-FU (5 µM) (**A′**) or paclitaxel (2–3 nM) (**A″**). Results were normalized to control EL treatments. The mean of triplicate measurements from a single trial ± SD are shown. (**B**) Colony formation assays were performed on BT549, and SK-OV-3 cells which were either left untreated (UT) or treated with BP1003 alone, or in combination with paclitaxel for 10 days. The mean of triplicate measurements from a single trial ± SD are shown (* = *p* < 0.05, ** = *p* < 0.01, *** = *p* < 0.001, **** = *p* < 0.0001).

At 200 μg/mL, BP1003 monotherapy decreased cell viability to ~70% (Figure 2A). 5-FU (5 µM) decreased cell viability to 60–70% (Figure 2A′). Combining these two drugs decreased cell viability to a greater extent: 30% viability in SK-BR-3 and SK-OV-3 cells, and 40% viability in BT549 cells (Figure 2A′). Similarly, BP1003 in combination with paclitaxel was more effective than either agent alone; the combination decreased viability to ~50% in SK-BR-3 cells and 30% in SK-OV-3 and BT549 cells (Figure 2A″).

Colony formation assays were also used to determine the effect of reduced STAT3 expression on cell proliferation. BP1003 reduced BT549 colony formation and density in a dose-dependent manner (Figure S2B). Treatment of BT549 or SK-OV-3 cells with 150 µg/mL of BP1003 reduced colony formation by ~45 and 20%, respectively (Figure 2B). Combining BP1003 with paclitaxel further decreased colony formation by 70% in BT549 and 50% in SK-OV-3 cells (Figure 2B).

These results show that BP1003 monotherapy significantly reduces the ability of cancer cell lines to proliferate in vitro as well as their ability to form and grow colonies. These effects were further enhanced when BP1003 was used in combination treatments with paclitaxel or 5-FU.

### 3.3. BP1003 Reduces Cell Migration

**Figure 3:**
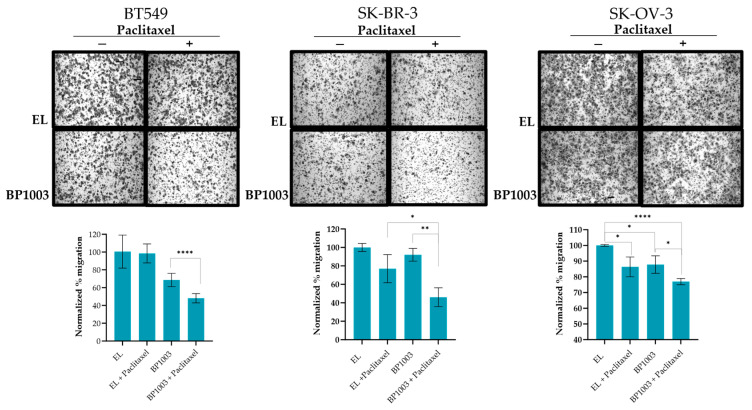
BP1003 reduces cell migration. Cell migration was investigated using transwell chambers. SK-OV-3, BT549, and SK-BR-3 cells, pre-treated with 200 μg/mL or BP1003 alone or in combination with paclitaxel (2 nM), were given appropriate times to migrate directly across the transwell membrane. After crystal violet staining, the percent area occupied by migrated cells was quantified through 3 sections of each membrane. Images were taken at 10× magnifications (* = *p* < 0.05, ** = *p* < 0.01, **** = *p* < 0.0001).

The effect of inhibiting STAT3 expression on the metastatic phenotype of cancer cell lines was investigated using trans-well migration. Treatment of BT549 cells with BP1003 resulted in a 30% reduction in cell migration compared to control EL treatment (Figure 3). Migration decreased by 13% in SK-OV-3 cells and was unchanged in SK-BR-3 cells. However, combination treatments of BP1003 and paclitaxel significantly decreased the migration of SK-OV-3 and SK-BR-3 cells by 25% and 55%, respectively. Combination treatments of BT549 cells further reduced cell migration to 48% (Figure 3).

### 3.4. BP1003 Interferes With Spheroid Formation and Growth

**Figure 4:**
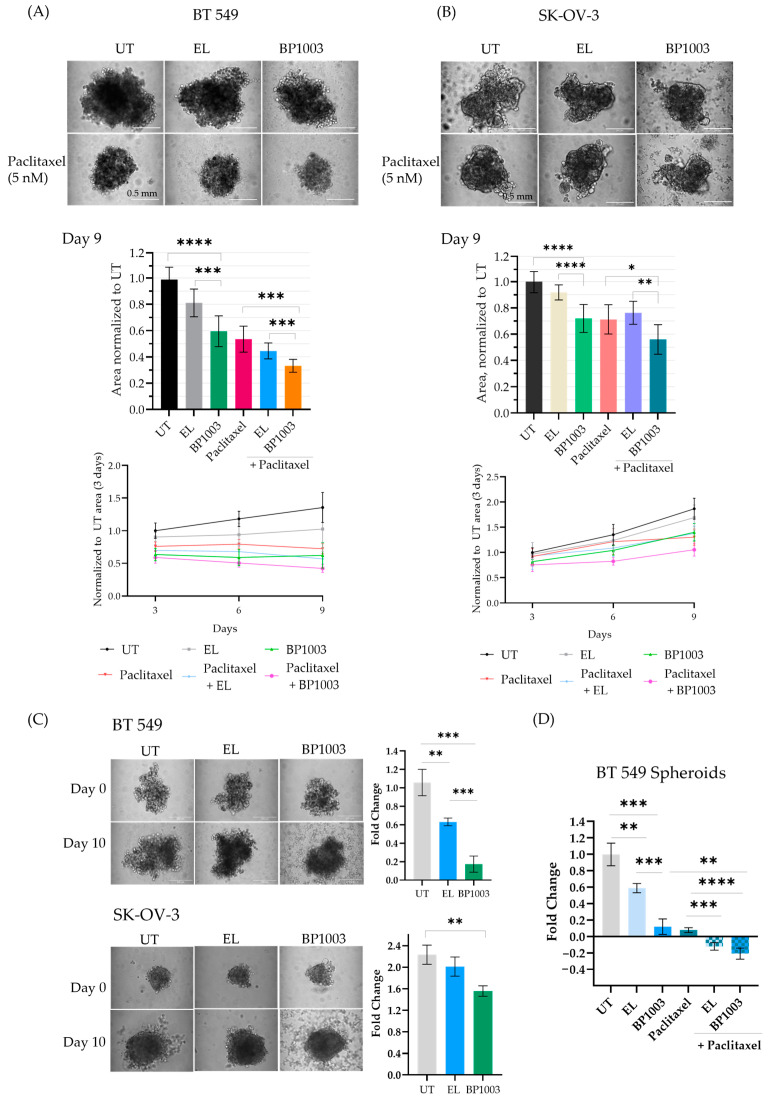
Effect of BP1003 and paclitaxel on spheroid formation and growth. 2D pretreatment of (**A**) BT549 and (**B**) SK-OV-3 cells with BP1003 (250 µg/mL) for 72 h, +/−paclitaxel treatment 24 h after plating in round bottom wells. The size and growth rate of the spheroids were monitored over 9 days. Images represent spheroids on the 9th day (scale bar of 200 µm) and bar graphs represent the area measured with ImageJ from a minimum of two independent experiments. Error bars represent the mean (*n* = 6) ± SD. (**C**) Treatment of pre-formed BT549 and SK-OV-3 spheroids with BP1003 (250 µg/mL) and (**D**) combination treatment of BT549 spheroids with BP1003 and paclitaxel reduced the fold change (y/x −1) of spheroid area after 10 days. Bar graphs represent the area measured using ImageJ from a minimum of two independent experiments. Error bars represent the mean (*n* = 6) ± SEM (* = *p* < 0.05, ** = *p* < 0.01, *** = *p* < 0.001, **** = *p* < 0.0001).

Experiments were undertaken to determine if BP1003 impacts the ability of BT549 and SK-OV-3 cancer cell lines to form spheroids. Pretreatment of BT549 and SK-OV-3 cells with BP1003 or paclitaxel reduced the size of the spheroids formed as early as 3 days (Figure S3A and Figure 4A,B). Combination treatment resulted in BT549 spheroids similar in size to BP1003 monotherapy treatments after 3 days but displayed a 70% decrease in size after 9 days (Figure 4A). After 9 days, BP1003-treated SK-OV-3 spheroids were 30% smaller than untreated spheroids and 54% smaller with paclitaxel combination treatments (Figure 4B). These results show that BP1003 compromises spheroid growth as a monotherapy and more significantly in combination with paclitaxel.

The effects of BP1003 on formed spheroids (3 days post plating) were then investigated. Spheroid growth was followed for an additional 10 days and the area of treated spheroids was normalized to that of untreated spheroids (Figure 4C). EL treatment led to a 0.6-fold increase in BT549 spheroid size while BP1003 treatment led to a 0.2-fold increase (Figure 4C). SK-OV-3 spheroids increased dramatically in size, with untreated spheroids being 2.1-fold larger after 10 days, EL-treated spheroids 1.9-fold larger, and BP1003-treated spheroids 1.7-fold larger (Figure 4C). Combination treatments once again further reduced spheroid size compared to single drug treatments. (Figure 4D). Similar results were obtained with larger spheroids, formed from a greater number of cells (Figure S3B,C).

Having established that the proliferation, colony growth, and spheroid formation of cancer cell lines were sensitive to BP1003, both as a single agent and in combination with chemotherapy, we determined whether BP1003 could affect tumor growth in ex vivo and in vivo models.

### 3.5. BP1003 + Gemcitabine Combination Treatment Results in Decreased PDAC PDX Tissue Slice Viability and Tumor Regression

A panel of 18 PDAC PDXs was used in an ex vivo live tissue sensitivity assay (LTSA) to study the activity of BP1003 alone and in combination with gemcitabine. Using previous defined criteria, tissue slice viability inhibition greater than 30% was considered a significant response [34]. Treatment of tissue slices with BP1003 alone significantly decreased the viability of 7 out of 18 PDAC PDXs by over 30% (*p* < 0.05) (Table 1). Gemcitabine treatments alone did not significantly affect any of the 18 tissue samples. BP1003 and gemcitabine combination treatments reduced tissue slice viability by over 30% in 11 PDAX PDXs and by over 50% in 5 out of the 18 PDAX PDXs (Table 1). Based on these results, PDAC-PDX 055 was selected to be used for in vivo studies due to its efficient growth in nude mice and its sensitivity to BP1003, gemcitabine and the synergistic effect of the BP1003 + gemcitabine combination.

**Figure 5:**
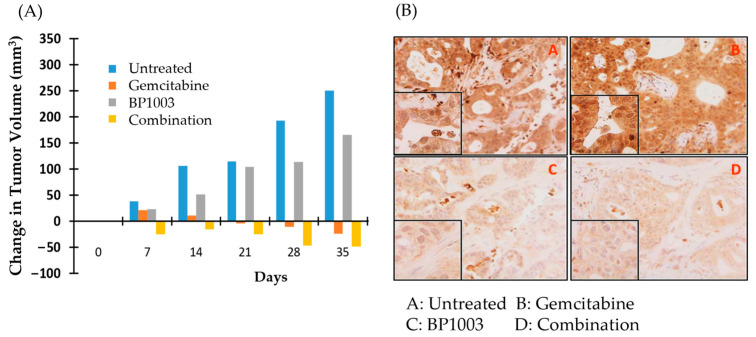
BP1003 and gemcitabine combination promoted PDAC PDX tumor regression. (**A**) Change in PDAC PDX tumor volume over time. Measurements taken every week, from the beginning of drug treatments to one week after the termination of drug treatments. (**B**) Histologic images of PDAC PDX tumor samples with STAT3 staining (10× magnification).

Nude mice implanted with PDX 055 xenografts were treated with BP1003 or gemcitabine alone, or in combination. Tumor volumes, measured weekly, revealed that gemcitabine monotherapy inhibited tumor growth and reduced tumor volume after 28 days. BP1003 monotherapy did not reduce tumor volume but decelerated tumor growth (Figure 5A). Combination treatments decreased tumor volume in just 7 days and to a greater extent than gemcitabine alone in 28 days. The anti-cancer activity of combination treatments was maintained beyond the 28-day treatment window after termination of treatments, indicating prolonged therapeutics effects. Immunohistochemical staining of tumors at the end of the study showed a dramatic reduction in STAT3 levels in tumor samples treated with BP1003 alone (Figure 5B, panel C). On the other hand, tumors treated with gemcitabine alone displayed an increase in STAT3 levels (Figure 5B, panel B); this effect has been observed in other studies and is suggested to be a potential contributor to gemcitabine resistance in pancreatic cancer cells [35,36]. Reduction in STAT3 levels was noted in tumors treated with the BP1003 + gemcitabine combination (Figure 5B, panel D). Having shown that BP1003 effectively inhibited the growth of PDX ex vivo and in vivo, we next investigated whether BP1003 could modulate immune cell function in the tumor microenvironment by inhibiting the polarization of monocytes toward anti-inflammatory, TAM-like M2 cells.

### 3.6. BP1003 Reduces M2 Polarization but Has No Effect on Previously Polarized Monocytes

**Figure 6:**
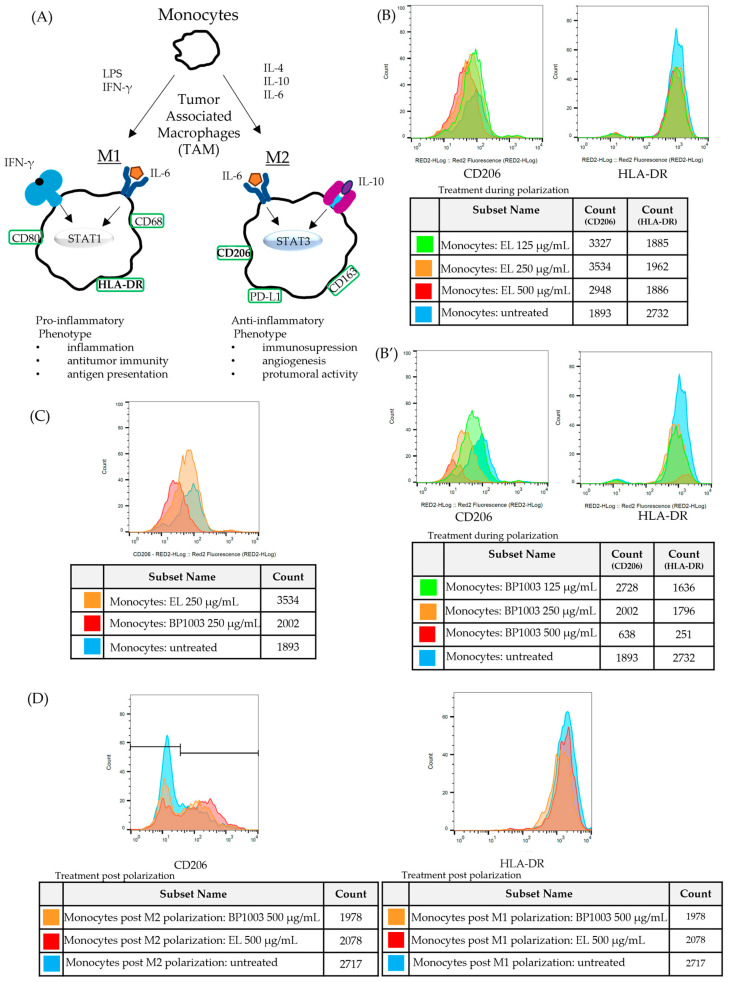
BP1003 reduces M2 monocyte polarization. (**A**) For in vitro polarization, monocytes were stimulated with LPS and IFNγ for M1 polarization and IL-4 for M2 polarization. (**B**–**D**) Representative histograms for expression of surface markers CD206 (M2) and HLA-DR (M1). (**B**,**B′**) To assess the effect of BP1003 and EL during polarization monocytes were treated with the indicated dilutions of BP1003 or EL at the same time as polarizing agents for 3 days. (**C**) BP1003 treatments at 250 µg/mL most efficiently decreased M2 polarization. (**D**) Neither M1 nor M2 polarized monocytes are affected by BP1003 or EL (500 µg/mL).

The progression of naïve, undifferentiated monocytes to monocytes/macrophages with either pro- (M1) or anti- (M2) inflammatory functions is driven by different stimuli and is associated with phenotypic changes that are distinctive between functional subsets. To determine, via phenotypic analysis, whether BP1003 has any effects on the polarization of monocytes, BP1003 was added to CD14+ monocytes cultures being treated with cytokine stimuli for M2 or M1 polarization. M2 macrophages were identified by the expression of the mannose receptor CD206 (Figure S4). Major histocompatibility complex (MHC) II cell surface receptor (HLA-DR) was used as a marker for M1 macrophages (Figure 6A).

Inclusion of EL in either M1 or M2 polarization cultures had no effect on HLA-DR or CD206 expression, respectively, and minimal effects on cell recovery (Figure 6B). On the other hand, BP1003 inclusion in the M2 polarization cultures reduced CD206 expression in a dose dependent fashion while having no effect on HLA-DR expression (Figure 6B′). Interestingly, when using BP1003 at 500 µg/mL, while M2 polarization was essentially blocked, cell recovery was also significantly reduced. The reduced cell recovery seen with 500 µg/mL BP1003 treatment of either M1 or M2 polarizing monocytes suggests the possibility of a non-specific cytotoxic effect on naïve monocytes (Figure 6B’). This cytotoxic effect was only seen at 500 µg/mL as the 250 µg/mL dose clearly reduced CD206 expression without reducing cell recovery (Figure 6C).

To determine if BP1003 could also affect the number of pre-established M2 macrophages, monocytes that had previously been polarized to M1 and M2 in culture were incubated with concentrations of BP1003 or EL for a period of time comparable to that used for cell treatment prior to polarization. For M1 cells there was a minor reduction in cell recovery for both the BP1003 and EL cultures at 500 µg/mL but no effect on expression of HLA-DR (Figure 6D). Similar results were obtained with M2 polarized cells; no appreciable effect of 500 µg/mL BP1003 was seen on CD206+ cells apart from a minor drop in cell recovery that was also seen for EL treated cultures. Notably, the reduced numbers of cells recovered was limited to the cells negative for CD206 (peaks to the left of the graph) (Figure 6D).

We conclude that BP1003 is highly effective at blocking the polarization of monocytes to an CD206+ M2 phenotype in the 500 µg/mL to 250 µg/mL dilution range in vitro. We do not know the fate of STAT3-blocked monocytes subjected to an M2 polarization stimulus. Although at 500 µg/mL cytotoxicity is detected, it is possible that some of this toxicity may be selective for cells that would otherwise polarize to M2. Previously polarized M2 macrophages are unaffected by BP1003 treatments, and, like M1 macrophages, they are more resistant than naïve monocytes to the cytotoxic effects of BP1003 at high concentrations.

## 4. Discussion

Our data show the therapeutic potential of BP1003 as it inhibits multiple tumorigenic processes, including cell viability, drug resistance, colony and spheroid formation, and migration, in different types of cancers. The effectiveness of BP1003 is likely due to its ability to decrease STAT3 levels, which leads to decreased expression of its downstream targets, such as vimentin and Bcl-2, which have both been associated with tumor aggression and poor prognosis.

BP1003 also exhibits immunomodulatory potential as a highly effective inhibitor of M2 monocyte polarization. The inclusion of BP1003 in cultures of CD14+ monocytes isolated from normal human peripheral blood and treated with the M2-polarizing cytokines M-CSF and IL-4 prevents their polarization to CD206+ M2, anti-inflammatory macrophages. In contrast BP1003 treatment has no effect on the polarization of naïve human monocytes to pro-inflammatory M1 macrophages. Our data also suggest that at relatively high concentrations BP1003 may have a selective effect on the viability or in vitro expansion of naïve monocytes, particularly if committed to an M2 lineage. While no effect on the phenotype of previously polarized monocytes was seen upon treatment with BP1003, based on the current data, we cannot speculate as to whether there is a functional effect on either cell population. Measurement of cytokine production or gene expression by cells would be required to determine if there is an effect on cell function. However, it is very likely that the inhibition by BP1003 of CD206 expression during polarization has a profound impact on the acquisition of M2 functions by the cells as mechanistic links between the expression of this molecule and cell function have previously been reported [37]. These results provide initial proof of the principle that BP1003 may be a highly effective inhibitor of M2 polarization and raise the question as to whether STAT3 activity is required to maintain M2 functionality after polarization and if it is required for the survival of naïve monocytes in culture.

The critical role of STAT3 in tumorigenesis makes it an ideal drug target. However, therapeutic inhibition of STAT3 has been very challenging because STAT3 is downstream of multiple tyrosine kinases and is regulated by a range of post-translational modifications and noncoding RNA. Extensive research has been devoted to developing drug candidates that can directly target STAT3. These candidates include DNA decoys, small molecules, and peptides that target the DNA- or SH2-binding domains of STAT3 [12,23,24,38,39,40]. Such approaches may not be entirely successful in cancer therapy because in addition to its canonical nuclear activity in gene regulation, STAT3 has non-canonical functions by regulating cellular respiration, energy production, and reactive oxygen species levels in the mitochondria [41,42,43].

ASO technology has the advantage of specifically blocking the synthesis of the targeted protein with more stability than peptides and less potential toxicity than small molecules [25]. There are many structure and sequence similarities among the seven STAT proteins. Sequence alignments reveal that STAT4 and STAT3 mRNA differ by 5 out of the 18 nucleotides at the BP1003 target site. Our data showed that this 5-nucleotide discrepancy is sufficient to make STAT3 a unique target of BP1003 as STAT4 levels were not reduced by BP1003.

Danvatirsen (IONIS-STAT3/AZD9150), a STAT3-targeting ASO that contains constrained ethyl-modified residues, was shown to be selective for STAT3 without affecting the expression of other STATs [44]. Clinical activity was seen in an early danvatirsen monotherapy study [44]. Recent studies exploring danvatirsen in combination with acalabrutinib in relapse/refractory diffuse large B cell lymphoma or danvatirsen in combination with durvalumab in refractory solid tumors demonstrate that the drug combinations are safe and tolerable but only modest clinical activity with limited durability was observed [45,46].

The neutral charged, hydrophobic P-ethoxy STAT3 ASO is efficiently incorporated in neutral DOPC liposomes. BP1003 is formulated with the same DNAbilize^®^ technology as Prexigebersen, an ASO that targets the Grb2 mRNA. Prexigebersen is currently being investigated in Phase II clinical trials for AML patients (NCT02781883) after promising clinical activity, and no dose limiting toxicities were observed in earlier Phase I trials [25,26]. Being both neutrally charged and non-immunogenic limit the interaction of BP1003 with charged plasma proteins, thereby increasing its stability and cellular uptake. BP1003 penetrates into PDAC PDXs, suppresses STAT3 expression, and induces tumor regression when combined with gemcitabine. BP1003 also selectively inhibits M2 monocyte polarization with consequences for tumor immunotherapy. The tolerance and efficacy of the DNAbilize^®^ technology in patients, and the multi-faceted inhibitory effects of BP1003, especially in combination therapies, are highly suggestive of its therapeutic potential against STAT3 in a range of solid tumors.

## Figures and Tables

**Table 1:** Ex vivo LTSA with BP1003 +/− gemcitabine on PDAC PDX tissue slices.

PDAC PDX	Decrease in Viability (% of Untreated)		
	Gemcitabine (10 µM)	BP1003 (60 µg/mL)	Gemcitabine + BP1003
102	0	11	17
124	7	14	16
032	0	15	18
053	14	16	28
147	10	17	43
118	0	19	48
070	0	22	26
155	10	24	26
110	9	26	40
059	5	27	26
162	0	29	53
055	15	31	49
176	11	35	56
030	5	35	48
213	11	35	43
045	12	46	56
179	0	47	52
113	9	58	55

## Data Availability

No new dataset was created. Data are contained within the article and the Supplementary Materials.

## Supplementary Materials

The following supporting information can be downloaded at: https://www.mdpi.com/article/10.3390/biomedicines12081901/s1, Figure S1: Time course STAT3 and target protein levels with BP1003 treatment; Figure S2: Cell viability and colony formation dose response to BP1003; Figure S3: BP1003 decreases spheroid size in combination treatments; Figure S4: M2 polarized monocytes express CD206.
