# Supplementary material for: BP1003 Decreases STAT3 Expression and Its Pro-Tumorigenic Functions in Solid Tumors and the Tumor Microenvironment
Source: Biomedicines. 2024 Aug 20;12(8):1901. doi: 10.3390/biomedicines12081901 (PMC11351911; doi:10.3390/biomedicines12081901)
Supplement: Supplementary file 1 [file biomedicines-12-01901-s001.zip › biomedicines-3114238-supplementary.pdf]

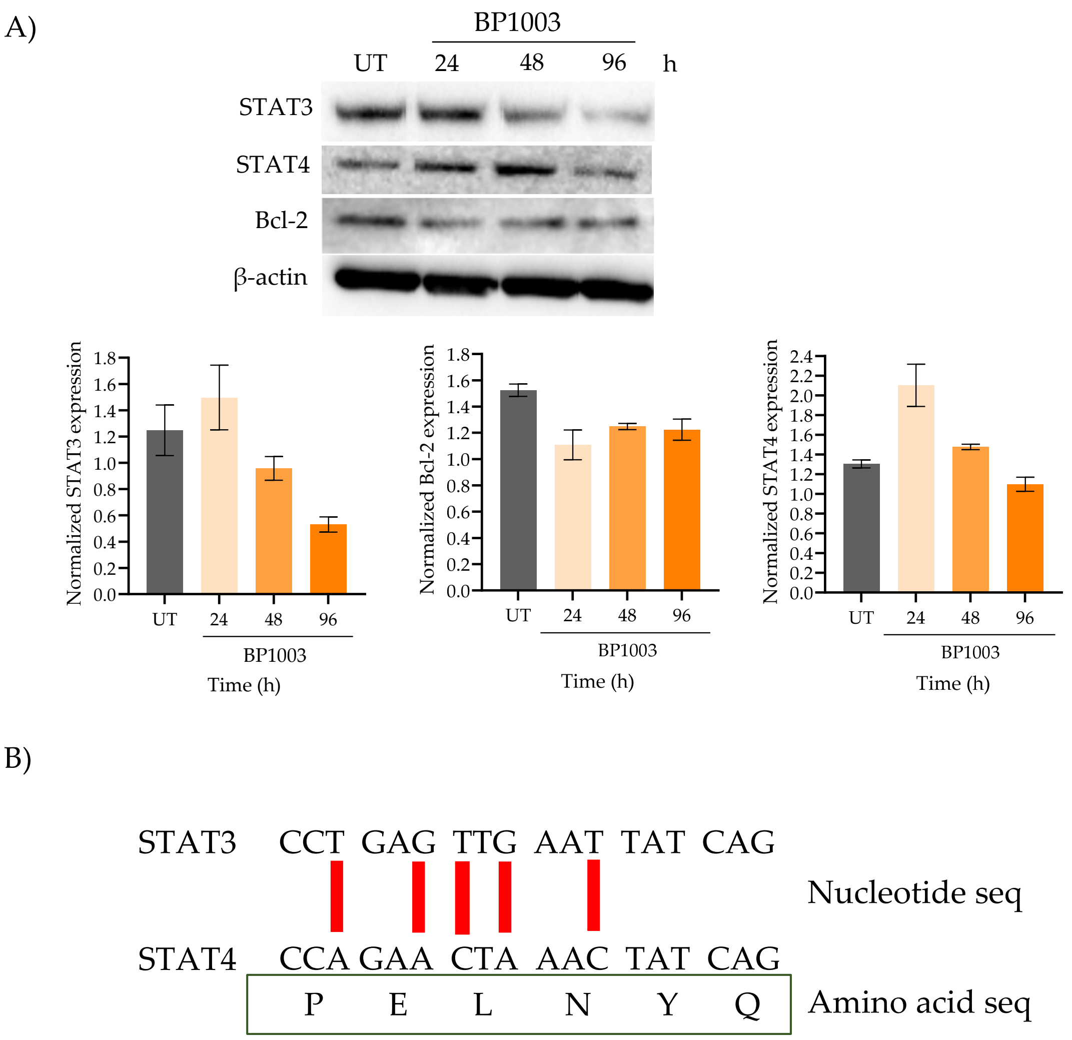

Figure S1. Time course STAT3 and target protein levels with BP3 treatment. **A**) Time course of BP1003 shows reduction of STAT3 and Bcl-2 over 96 h in SK-OV-3 cells. STAT4 levels are unchanged. Graphs represent quantification of protein bands. **B**) Sequence similarity between STAT3 and STAT4 at BP1003 target site.

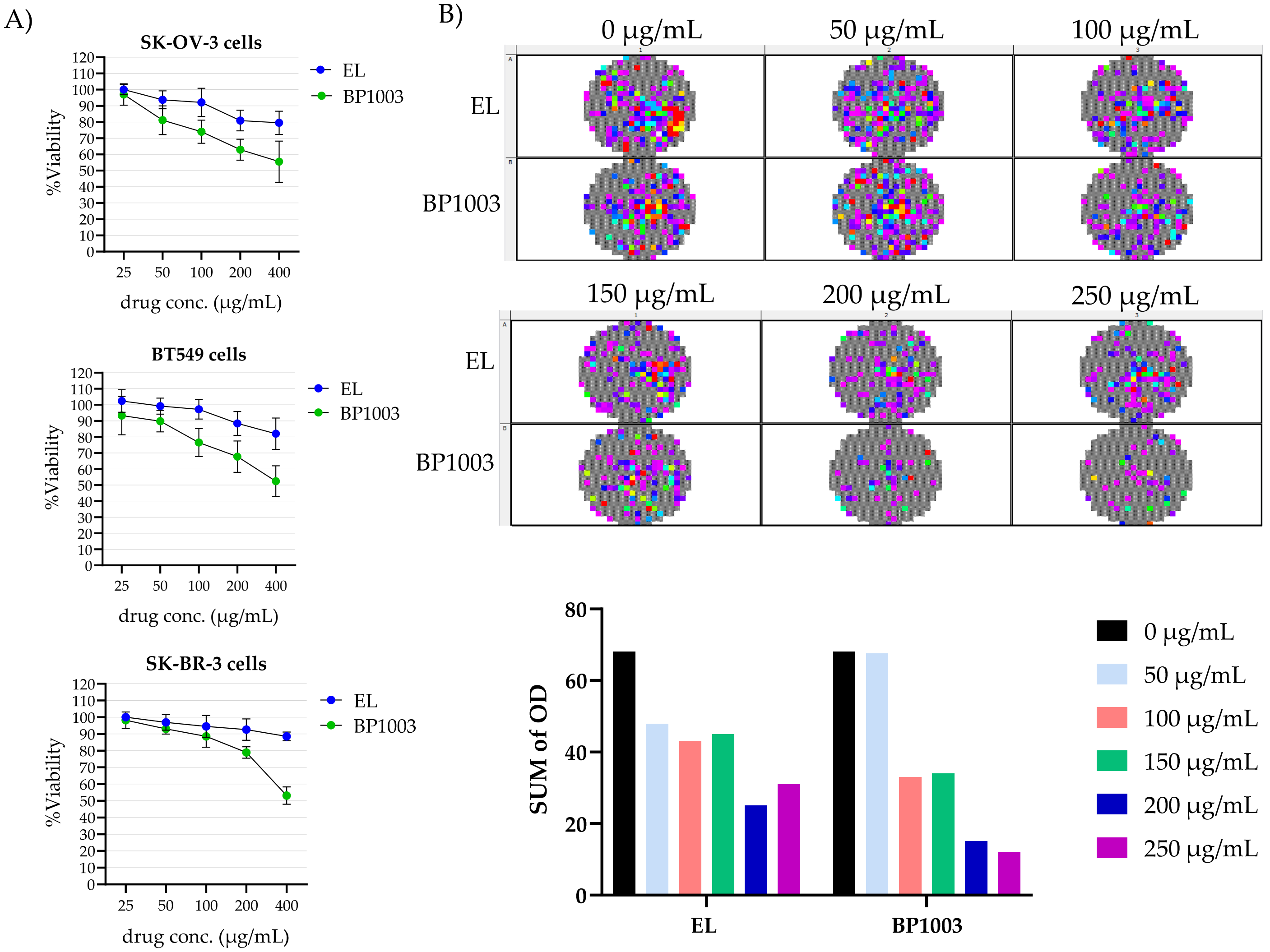

Figure S2. Cell viability and colony formation dose response to BP1003. **A**) Dose–response curves of SK-OV-3, BT549 and SK-BR-3 cells to BP1003 or EL. The mean of triplicate measurements from a single trial are shown. **B**) Dose response of BT549 cell colony formation. Images represent the optical density (OD) of the crystal violet staining obtained from the FLUOstar microplate reader which is quantified in the graph.

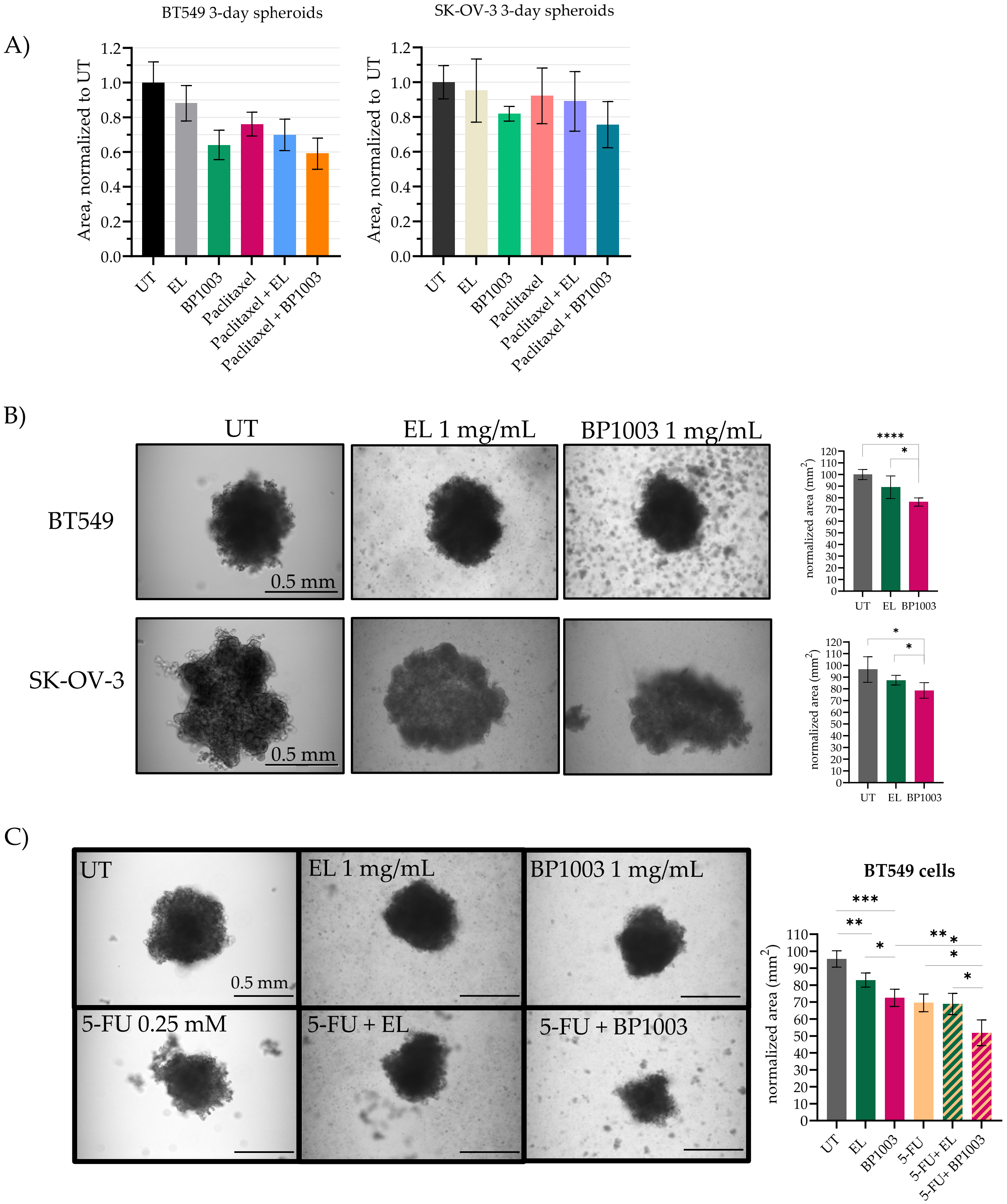

Figure S3. BP1003 decreases spheroid size in combination treatments. **A**) Size of 3-day old spheroids formed from BT549 and SK-OV-3 cells pretreated with BP1003 (250 μg/mL), +/- paclitaxel (5 nM). **B**) The effect of 1 mg/mL BP1003 on large spheroids, formed from 4000 BT549 or SK-OV-3 cells after 3 days of treatment. **C**) Large BT549 spheroids sequentially treated with 0.25 mM 5-FU and 1 mg/mL of BP1003 for 4 days. Error bars represent the mean ± SD. (* = $p < 0.05$, ** = $p < 0.01$, *** = $p < 0.001$).

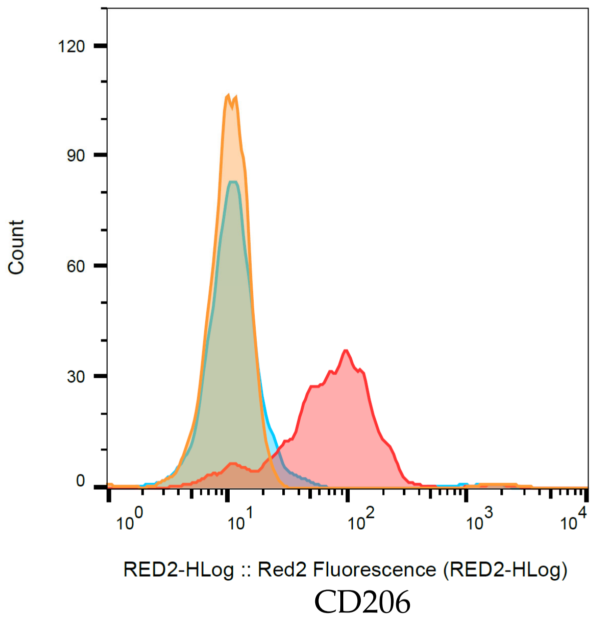

	Subset Name	Count
	Monocytes: untreated	2804
	Monocytes: M2 polarized	1893
	Monocytes: M1 polarized	2485

Figure S4. M2 polarized monocytes express CD206. For *in vitro* polarization, monocytes were stimulated with LPS and IFNγ for M1 polarization and IL-4 for M2 polarization. Representative histogram for expression of surface markers CD206 (M2).
